# Effect of sulfur doping of zinc-imidazole coordination polymer (ZnIm CP) as a novel photocatalyst for degradation of ionic dyes

**DOI:** 10.1186/s13065-022-00877-z

**Published:** 2022-11-04

**Authors:** Mostafa Mohsen, Ahmad Baraka, Ibrahim Naeem, Hesham Tantawy, Mohamed Awaad, Osama Abuzalat

**Affiliations:** Chemical Engineering Department, Military Technical College, Cairo, Egypt

**Keywords:** Photocatalysis, Modification, Non-metal doping, Visible, UV–VIS

## Abstract

Zinc-Imidazole coordination polymer (ZnImCP) was simply synthesized hydrothermally at relatively low temperature (70 °C) from zinc acetate and imidazole. ZnImCP was treated by sulfide solution to produce sulfur-doped samples (S-ZnImCPs). Structures of the synthesized ZnImCP and S-ZnImCPs were characterized through FTIR, PXRD, and, Raman, SEM/EDX, N_2_-BET, UV–VIS DRS, and pH_pzc_ analyses. The photocatalytic performances of pristine CP and sulfur modified CPs under visible and ultra-violet irradiations for degrading the cationic methylene blue (MB) and the anionic methyl orange (MO) were investigated considering different initial pH values 4, 7 and 10. Under visible light, the results indicate that these CPs display considerable photocatalytic degradation towards the cationic MB for the initial pH 4 and 7 where degradation increases with sulfur content. While under ultra-violet, results indicate considerable photocatalytic degradation towards both dyes for the initial pH 7 and 10 where degradation increases with sulfur content which indicates the gainful of non-metal dopping. The buffering nature of CPs and the type of radiation considering determined band-gap values effectively influence the degradation mechanisms.

## Introduction

Coordination polymers (CPs) and the sub-category metal–organic frameworks (MOFs) are hybrid materials constructed from metal nodes and organic linkers. These materials have recently received more attention as photocatalysts by the virtue of their unique properties of designable structure and large surface area and/or photo-responsive [1]. The tunable band-gap of CPs/MOFs encourages studying different structures for photocatalysis, especially photo-catalytic degradation of organic wastes in aqueous media and many researchers have developed novel CPs and MOFs for photocatalytic degradation of organic dyes. To name a few, Liming Fan et al*.* synthesized five different coordination polymers based on rigid tetracarboxylic acids and imidazole linkers using Cu, Ni, Co, and Zn as nodes and the produced CPs accomplished degradation efficiency up to 97.3% for methylene blue (MB) after 2 h, in general [2]. Cui et al. used rigid and semi-rigid bis(imidazole) ligands to generate two Co(II) based CPs. The synthesized CPs, under UV–Vis irradiation, showed degradation efficiencies of 91.4% with MB and 82% with congo red within 120 min [3]. Yang et al. synthesized some 3D supramolecular Cd(II) CPs based on aromatic polycarboxylate and bis(imidazole) ligands and a degradation efficiency of 85% for MB was reached within 120 min [4].

CPs based on *d*^10^ metals, such as Zn^2+^ and Cd^2+^, are capable to deliver an assortment of structures due to their flexible coordination environments that come from the absence of ligand field constraints. Moreover, The forbidden *d*–*d* transitions should allow CPs for luminescent emission and/or some other de-excitation mechanisms [5, 6]. Metal ions with *d*^10^ configuration (as nodes) and conjugated organic ligands (as linkers) are an appropriate choice to assemble CPs/MOFs as photoactive materials, and hence have the potential of being photocatalysts [7, 8]. Moreover, the *d*^10^ ions, such as Zn(II), have the least suitability towards oxidation/reduction, i.e. shows chemical stability, besides the hydrostability which gives the candidate to apply under hydro-redox conditions such as that of photocatalysis [9–11]. Concurrently, Imidazole derivatives, as a conjugate system, have proved to be suitable selection, as ligands, for constructing CPs/MOFs to apply as photocatalysts [3]. To our knowledge, however, the parent imidazole has been rarely utilized for constructing CPs/MOFs for the sake of being applied as a photocatalyst [12].

Hence, in the present work, Zn(II)/Imidazole coordination polymer (ZnImCP) has been synthesized and characterized and then assessed for its photocatalytic activity for degrading two dyes, the cationic methylene blue (MB) and the anionic methyl orange (MO). The aim of using different ionic types of dyes (cationic and anionic) is to consign the correlations of degradation profiles of the dyes with the character of ZnImCP as a catalyst and the applied conditions, specifically the light type and the applied initial pH.

Doping classical photocatalysts, e.g. TiO_2_, with metal or nonmetal ions has proved to be efficient to enhance photocatalysis [13]. Enhancement could arise from either of the following: (i) contraction of the band-gap [14], (ii) creation of intermediate energy levels, (iii) oxygen vacancies formation, and (iv) electron trapping [15]. likewise, CPs doping has been a subject matter where doping by metals to enhance photocatalysis is a well-known approach and has been widely covered [16, 17]. Still, exploring doping by non-metals is very rare [18, 19].

Hence, also in the present work, doping of ZnImCP with the non-metal sulfur is considered to investigate its effect expecting photocatalytic degradation enhancement and this should be an early effort, to the best of our knowledge, to deal with this subject. Doping by non-metal should be effective at enhancing photocatalysis performance due to expected bonding within the CP matrix [17, 20]. The intended selection of sulfur to dope by is based on the fact that it ready bonds to Zn(II). Accordingly, sulfur modified samples were also synthesized, in a simple way, to make use of the created d Zn–S bond to enhance photocatalysis [21]. Degradation experiments, for MB and MO, were performed under Vis-light and UV-light considering different initial pH levels [22]. Analogous photolysis degradations, i.e. applying lights without catalysts, were also performed for comparison and to assist suggested photocatalytic degradation mechanisms [23, 24]. Generally, it has been found that visible light resulted in considerable photocatalytic degradation of MB for acidic and neutral solutions where degradation increases with sulfur content. Meanwhile, ultra-violet resulted in considerable photocatalytic degradation towards both dyes for the neutral and basic solutions where degradation increases also with sulfur content. Discussion of degradation mecahnisms points to the importance of the buffering nature of CPs. The type of radiation consideing determined band-gap values effectively influence the degradation mechanisms as well. S-ZnImCPs should enhance the photocatalytic activity due to the incorporation of sulfide (sulfur atom) into ZnImCP structure.

## Experimental

### Hydrothermal Synthesis of pristine and sulfur modified ZnImCPs

To synthesize the pristine ZnImCP, imidazole solution (extra pure, Oxford, 2 mmol, 136 mg/10 ml DI water) and ammonia solution (1 ml, 33%, Piochem) were added together in a 100 ml Pyrex glass reactor. The mixture was left for 10 min to allow effective deprotonating of imidazole by ammonia. Ammonia (p*K*_a_ = 37) is a suitable deprotonating agent for imidazole (p*K*_a_ = 14.5) converting N of pyrrole ring to a lewis base site being ready for coordination which promotes imidazole for bidentate coordination with metal ions [12, 25]. Zinc acetate solution (Sigma, 1 mmol, 219 mg/10 ml DI water) was then added to the imidazole solution where immediate light turbidity eminences. The reactor was tightly closed and placed into a pre-heated oven at 70 °C where ZnImCP precipitate was observed to continually precipitate. After about two hours, no further precipitation was observed. Precipitated ZnImCP was collected and washed several times using warm DI water. Finally, ZnImCP was dried at 80 °C, and then stored for the afterward characterizations, modification by sulfur and photocatalytic degradation experiments.

Sulfur modified samples, S-ZnImCPs, were simply prepared via treating the pristine sample, ZnImCP, with solutions of different concentrations of sodium sulfide, Na_2_S. Treatment was performed by soaking each sample (100 mg) in Na_2_S solution (20 ml) of specified concentration for 24 h at room temperature, Table 1. This treatment aims to dope the pristine ZnImCP with non-metal sulfur. After treatment, samples were collected, washed several times using warm DI water and then dried and stored for the afterward characterizations and photocatalytic degradation experiments.

**Table 1:** Symbols of S-treated sample

Pristine Sample	Sulfide treatment	Symbol
ZnImCP	0 ppm	ZnImCP
	25 ppm	25S-ZnImCP
	50 ppm	50S-ZnImCP
	100 ppm	100-ZnImCP

### Characterization of the synthesized CPs

For chemical structure investigation, imidazole, ZnImCP and S-ZnImCPs were analyzed by FTIR spectroscopy (standard KBr pellet method, recording range: 400–3500 cm^−1^, 100 scans, and resolution of 4 cm^–1^, Jasco FT/IR 4100) and Raman spectroscopy (Sentera, Bruker, Germany, recording range: 400–3500 cm.^−1^). The crystalline nature of all CPs was studied by PXRD (Shimadzu XD-l) and their patterns were exported to the phase identification software (QualX, version 2.24) which uses the crystallography open database (COD) for search match. Surface morphologies of all CPs were determined using SEM (Zeiss EVO-10 microscopy) with making use of the coupled EDX to explore the elemental compositions of samples. The surface area of pristine ZnImCP was determined by applying standard N_2_-BET analysis (N_2_ adsorption, NOVA Station A) [12].

UV–VIS DRS equipped with the integrating sphere accessory for diffuse reflectance (Jasco V 530 spectrometer, Japan) was applied and spectra were recorded for all CPs. From these spectra, band-gap values, *E*_g_, were calculated using Tauc’s plot, (F.(*hν*)).^0.5^ versus *hν* where F = (1 − *R*)/(2R), *R* is reflectance, *h* is Plank’s constant, and *ν* is the frequency [4, 26]. For thermal stability investigation, TGA analyses were applied for all CPs (TGA discovery, TA instruments, USA) where 1 mg of each CP was heated at 20 °C/min from about 27 °C up to about 700 °C under N_2_ atmosphere (30 ml/min) [20, 27].

CPs stability in aqueous media is an important criterion regarding photocatalytic degradation applications in water. Many CPs are not that stable in aqueous media and therefore are not suitable for water treatment [10]. Hence, two tests were performed to evaluate the stability of the pristine ZnImCP: mass loss measurement and FTIR analysis. For the mass loss test, a specified amount of ZnImCP (100 mg) was soaked in water (100 ml) for 24 h at room temperature and then ZnImCP sample was separated, dried and weighed to determine the mass loss percentage.

The pH of point of zero charges (pH_pzc_) of the pristine ZnImCP has been also determined by the electrochemical method reported by S. Altenor et al. [28, 29]. The measures of pH values have been performed using pH-meter 720 (WTW-Inolab, Gemini BV).

### Photocatalytic activity Measurements

The photocatalytic activities of ZnImCP and S-ZnImCPs were evaluated by recording the degradation percentages of two different types of dyes: MB (cationic) and MO (anionic). The irradiation process was achieved by using Vis-lamp (35 W, λ = 400–800 nm) and UV-lamp (24 W, 254 nm) [30, 31]. It is important to mention that all degradation experiments have been conducted without the use of the regularly applied auxiliary oxidant, H_2_O_2_ which is an advantageous stance when these materials become practically applicable. In a typical experiment, the CP sample (50 mg) was placed in a beaker (100 ml) containing an aqueous solution of MB or MO (25 ml, 5 ppm) and the beaker was left for 60 min in dark to guarantee the establishment of adsorption/desorption equilibrium, if present. Solutions were brought into shaking setup (orbital shaker: 100 rpm) adapted with top-source applied light from a fixed distance. Periodically, a 3 ml sample of the solution was withdrawn to measure the remnant concentration of the dye using UV–Vis spectrophotometer (Agilent 60 Jasco V 530 spectrometer, Japan) at 664 and 465 nm for MB and MO, respectively. For both the dyes, different degradation experiments were performed at different initial pH levels: 4, 7, and 10. Photolysis experiments for these dyes were also performed under the same conditions. Degradation efficiency, *D*%, was calculated as the relation: *D*% = ((*C*_o_-*C*_t_)/*C*_o_) × 100 where *C*_o_ and *C*_t_ represent initial and remnant (at a certain time *t*) concentrations, respectively.

The re-use of CPs for degradation was also examined by performing three successive degradation experiments using the same CP sample under the same conditions. CP sample was recovered after each experiment and DI water-washed several times without any additional treatment, and then has been dried (at 80 °C for 6 h) and applied for the subsequent experiment under the same conditions. *D*% values were recorded for the three experiments.

## Results and discussion

### Characterization of the synthesized CPs

#### FTIR and Raman analysis

**Fig. 1:**
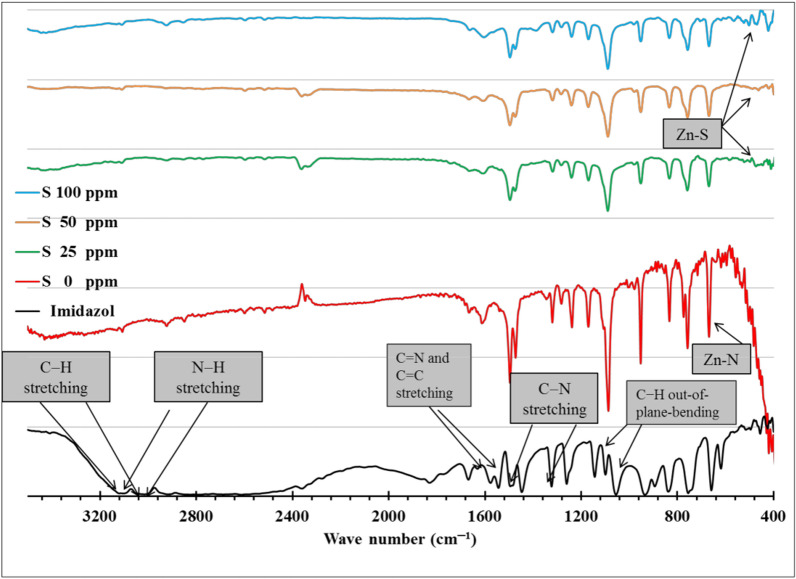
FTIR spectra of imidazole, ZnImCP and S-ZnImCPs

FTIR spectra of imidazole and ZnImCP and the modified S-ZnImCPs are shown in Fig. 1. Imidazole spectrum illustrates its known characteristic peaks: C − H stretching vibrations in the regions 3090–3140 cm^−1^, and 2900–2950 cm^−1^, and the N − H stretching vibrations cover the broad region from 2500–3000 cm^−1^ through two characteristic peaks at 2901 and 2995 cm^−1^ [32–34]. Besides, C − N, C = N, and C = C stretching are present and assigned by arrows as shown in the figure. Further, the obvious peaks at 1457 and 1365 cm^−1^ are assigned to C − N stretching mode [33, 35].

The two peaks appearing at 2900 and 3095 cm^−1^ within the spectrum of imidazole (those of C − H and N − H, respectively), disappeared from the spectrum of ZnImCP which indicates developing a change in the structure of imidazole with an increase in rigidity due to the coordination with a metal ion, as nodes, and deprotonation of N − H. The peaks of C = N, and C = C stretching of imidazole have disappeared as well from the spectrum of ZnImCP as imidazole reacts almost completely with Zn, confirming the alteration of imidazole structure due to the deprotonation process and the formation of coordination bonds with Zn. In addition and of importance, the coordination peak related to Zn − N bond can be observed in ZnImCP and S-ZnImCPs spectra at 667 cm^−1^ [36].

The obvious C − N stretching peaks at 1457and 1365 cm^−1^ in imidazole have been shifted to 1491 and 1367 cm^−1^ in ZnImCP spectrum which indicates that imidazole is still present in the new product. The stretching modes of C = C and C = N of imidazole shifted from 1540 and 1569 cm^−1^ into two characteristic peaks at 1585 and 1610 cm^−1^ in the ZnImCP spectrum. Such observed shift is related to a higher energy band inferring an increase of imidazole ring coherence owing to the formation of novel coordination chains that affect the ring rigidity [33, 34, 37]. The C − H out-of-plane-bending sharp peaks appearing at 1048 and 1095 cm^−1^ in imidazole have been shifted to 1086 cm^−1^ in the ZnImCP which manifest the existence of intact imidazole in the structure [34, 38].

The appearance of new weak peaks at 495 and 529 cm^−1^ in S-ZnImCPs spectra may indicate the presence of Zn–S bond in the S-ZnImCPs structures. The peaks became stronger at 100 ppm as sulfur content increased [39, 40]. It could be possible to hypothesize that sulfur (a strongly negative ion with lone pairs) coordinates with Zn(II) nodes by the use of an unsaturated metal site within ZnImCP structure. The impact of this hypothesis is that such Zn–S coordination bond could be very similar to that of ZnS, the known photocatalyst. Hence, this picture gives a structure of S-ZnImCPs that comprises some ZnS moieties. Accordingly, these ZnS moieties may support the photocatalysis behaviour of S-ZnImCPs.

The three spectra of S-ZnImCPs are very similar indicating that different S-treatments, due to concentration, did not affect the whole chemical structure. Almost all peaks of S-ZnImCPs spectra are in the same positions as the corresponding those of ZnImCP which indicate no major change in chemical structure, compared to ZnImCP upon S-treatment.

**Fig. 2:**
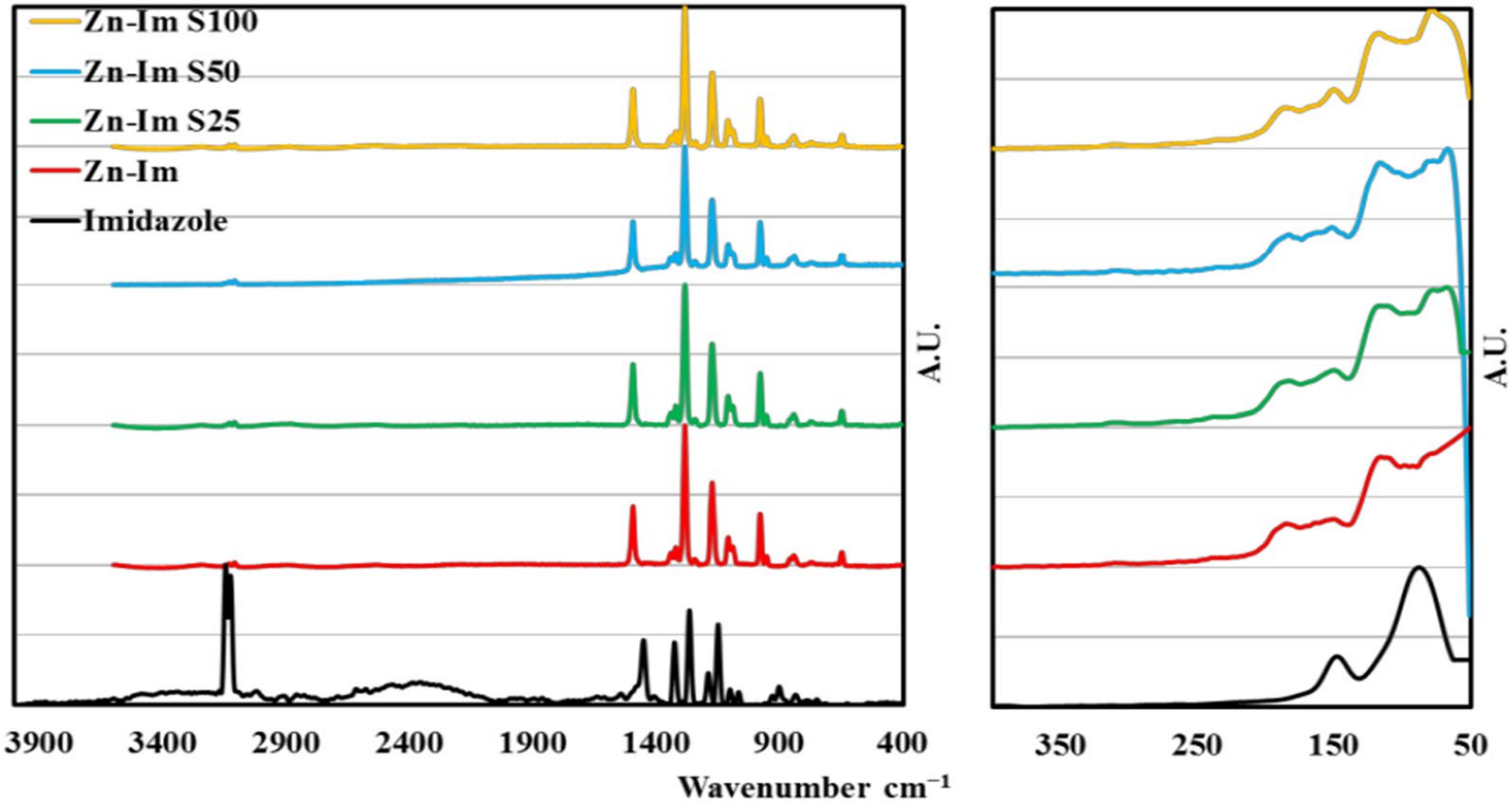
Raman spectra of imidazole, ZnImCP and S-ZnImCPs

Raman spectra of imidazole, ZnImCP and S- ZnImCPs are shown in Fig. 2. The first region, below 200 cm^−1^, is usually dedicated to the whole lattice vibrations of the tested material [41–43]. The appearance of characteristic peaks in this region should indicate the crystalline nature of the sample. As the figure depicts, imidazole has a set of two characteristic peaks in this region at 85 and 144 cm^−1^ [41, 42, 44]. Meanwhile, ZnImCP has a different set of three obvious peaks at 106, 136 and 181 cm^−1^ [45]. Two of these peaks are close to that of imidazole, 106 and 136 cm^−1^, in addition to a new one appearing at 181 cm^−1^. These results emphasize the presence of an imidazole molecule in the structure of pristine ZnImCP. The shifts of ZnImCP peaks to a higher energy band in addition to the newly appeared peak should be related to the formation of a new product containing an imidazole ring with new coordination bonds causing higher energy absorption. Importantly, the spectra of S-ZnImCPs comprise the same set of peaks of ZnImCP, however, in addition, an obvious extra peak at about 63 cm^−1^ emerged and should be related to the proposed sulfur bonding. In fact the observed new 63 cm^–1^ peak should be related to the inclusion of sulfur in the pristine CP matrix. i.e. presence of sulfur affect the whole lattice vibrations.

The second region of a Raman spectrum covers the range from 200 to 1800 cm^−1^ and considered as the fingerprint band. In this band, imidazole has four strong peaks at 1146, 1266, 1321 and 1447 cm^−1^ [46]. The weak peaks at 1057 and 1094 cm^−1^ should be assigned to the in-plane C − H bending deformation [46–48]. These two weak peaks show a shift to 1065 and 1089 cm^−1^, respectively in the new product, ZnImCP, due to the rigidity of the structure because of coordination. The strong peak appearing at 1266 cm^−1^ in imidazole spectrum also shows a shifte to 1281 cm^−1^ in the new product confirming the previous conclusion [46, 47, 49]. The strong peaks at 1146, 1321 and 1447 cm^−1^ are assigned to ring stretching and show shiftes in the new product to higher energy peaks at 1167, 1324 and 1488 cm^−1^, respectively due to the same reason for shifting the C − H peak [46, 47, 49]. The peak at 1182 cm^−1^ assigned to δ(NH) in-plane deformation has disappeared, in the new product indicating deprotonation. The appearance of a new small peak at 183 cm^−1^ strongly indicates coordination bonding between imidazole and Zn to N atom after deprotonation [46, 47, 50]. The vanishing of the two strong imidazole doublet peaks at 3123 and 3142 cm^−1^, assigned to C − H stretching, in the new products together with the appearance of the new strong peak at 973 cm^−1^ supports the formation of the coordination compound [46, 48, 51, 52].

#### PXRD analysis

**Fig. 3:**
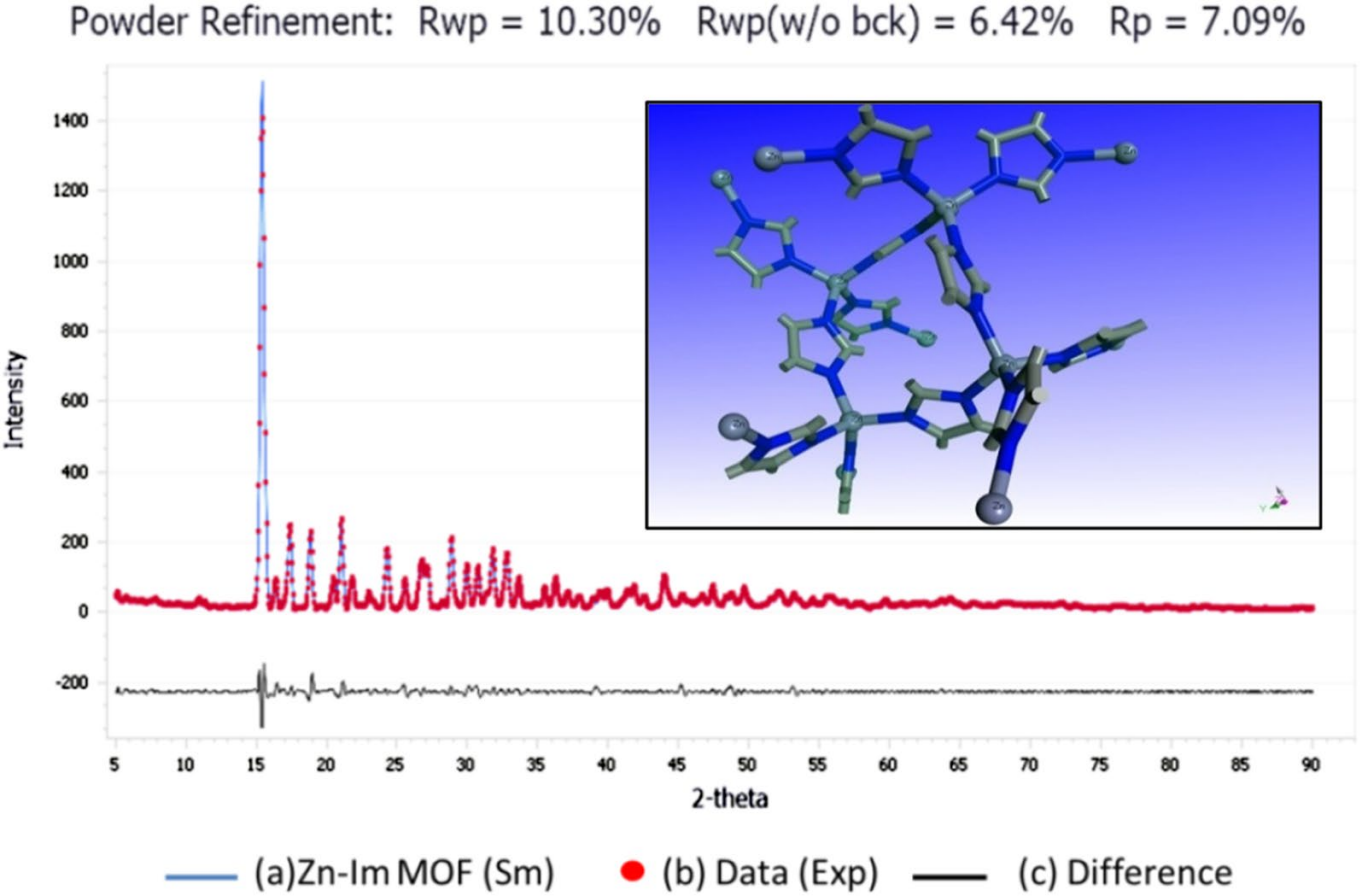
PXRD spectrum: **a** experimental; **b** simulated; and **c** difference. Inset: Proposed chemical structure of ZnImCP

Figure 3 shows the experimental PXRD pattern of ZnImCP accompanied by the simulated one. The comparison of the experimental PXRD pattern with the simulated one of the proposed structures, revelas obviously that the two patterns are very similar and highly coincide. This comes from the lower value of the weighted profile R-factor (R_*wp*_ = 10.30%), which is a determination of the degree of matching between the experimental pattern and the simulated one. The inset in the figure shows the proposed structure.

To resolve the structure of the synthesized ZnImCP, its PXRD pattern was exported to the phase identification software (QualX, version 2.24) which applies the crystallography open database (COD) for search match. Search match resulted in the zinc-imidazolate structure, (chemical formula: C_12_H_12_N_8_Zn_2_), with the figure of merit value of 0.82. The cif file of the compound was imported from Cambridge Crystallography Data Centre (CCDD) and additionally refined against the experimental PXRD spectrum via Materials Studio software (Biova, USA) to extract the experimental parameters using Pawley refinement. Table 2 gives the parameters of ZnImCP. The structure cell is tetragonal with a volume of 6788.81 A^3^ and it crystallizes in the I 41 C D space group. The detrmined formula unit of the compound is [Zn(Im)_2_]_n_ and there is sixteen formula unit in the cell. Table 3 lists the experimental parameters of PXRD analysis. Figure 3(inset) demonstrates the chemical structure of the 2D synthesized ZnImCP where Zn atoms are tetrahedrally coordinated to four imidazole rings validating the phenomenon that Zn(II) metal usually coordinates with 4-coordination number giving a tetrahedral geometry [53]. Clearly, ZnImCP is highly crystalline due to the sharp peaks of its spectrum. Benficially, this high crystallinity assists charge migration towards the surface upon excitation, i.e. less e^–^/h^+^ recombination phenomenon.

**Table 2:** Structural parameters of ZnImCP

Parameter	Value	Parameter	Value
Lattice type	Tetragonal	Space group	I 41 C D
a	23.36	Cell volume (A^3^)	6788.81
b	23.36	Density (g/cm^3^)	1.56
c	12.4	2ϴ range (degrees)	5.02–89.98
α	90.0	Step size (degrees)	0.04
β	90.0	Number of reflections	734
γ	90.0	Final R_p_	7.09%
Z	16	Final R_wp_	10.30%

**Table 3:** PXRD experimental parameters

FWHM	
U	1.06144 ± 0.04530
V	− 0.43825 ± 0.02825
W	0.14136 ± 0.00421
Profile function: pseudo-voigt	
NA	0.00000 ± 0.01133
NB	− 0.00000 ± 0.00040
Line shift instrument geometry: bragg–brentano	
Zero point	0.17007 ± 0.00172
Asymmetry correction: berar-baldinozzi limit: 30 (degrees)	
P1	− 0.01770 ± 0.03911
P2	0.17957 ± 0.00815
P3	0.11348 ± 0.07320
P4	− 0.38424 ± 0.01747

**Fig. 4:**
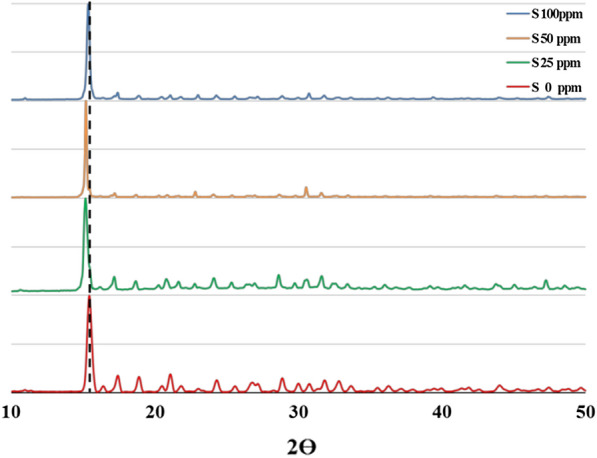
PXRD patterns of ZnImCP and different S-ZnImCPs

Figure 4 shows PXRD patterns of ZnImCP and S-ZnImCPs. ZnImCP and S-ZnImCPs which have very similar PXRD patterns. S-ZnImCPs samples are also of high crystallinity as indicated by their sharp peaks. The main peak of ZnImCP (2θ = 15.3) has slightly shifted to a lower value (2θ = 15.1) and retains its height in patterns of S-ZnImCPs indicating that the main matrix of CPs is intact after S-treatment. The other smaller peaks became less intense yet retain their positions and this should be also a sign of successful S-treatment. FTIR, Raman and PXRD (to less extent) analyses well suggest the successful chemical incorporation of sulfur in ZnImCP matrix, at least superficially [54].

#### SEM/EDX analysis

**Fig. 5:**
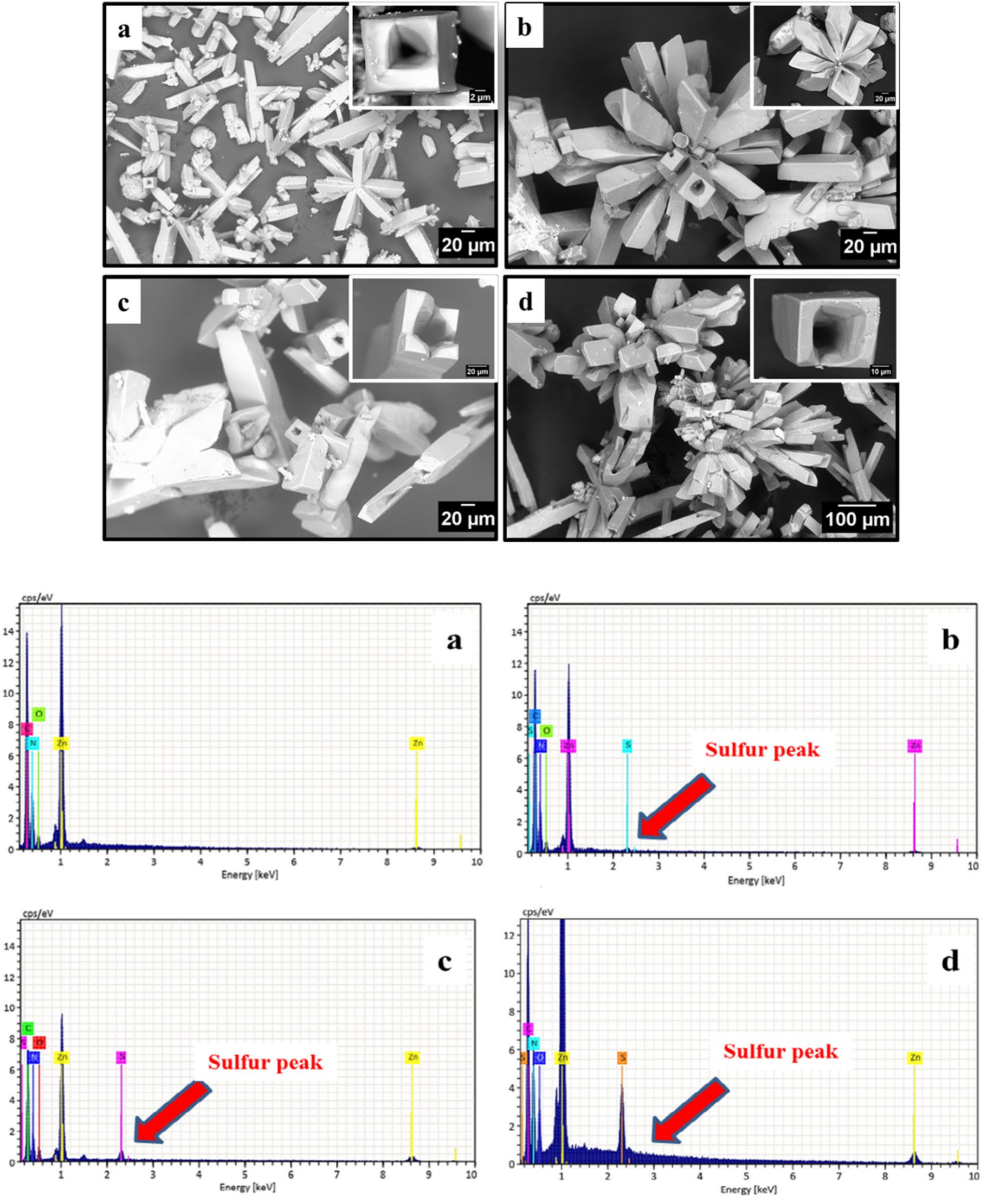
SEM images and corresponding EDX spectra below of **a** ZnImCP, **b** 25S-ZnImCP, **c** 50S-ZnImCP, **d** 100S-ZnImCP

Figure 5 shows the SEM images and the corresponding EDX spectra of ZnImCP and S-ZnImCPs. The images show that all samples have particles of the same shape being regular-ribbed and, interestingly, with longitudinal holes. According to EDX spectrum of ZnImCP, the elemental composition (% atom) is: Zn (5.5), C (55), N (35) and O (4.18) and no other elements were detected which indicates the purity of samples. The elemental ratios can be given as Zn(1): C(10): N(7), excluding O, and accordingly the carbon to nitrogen ratio in ZnImCP is about 1.57 which is similar to that of the imidazole molecule, that is 1.5. This suggests the intact presence of imidazole in structures of ZnImCP. For S-ZnImCPs, sulfur content increases gradually: Zn-Im(0 ppm S) < Zn-Im(25 ppm S) < Zn-Im(50 ppm S) < Zn-Im(100 ppm S). No other elements were detected which indicates the purity of the samples.

#### BET analysis

The BET analysis revealed that ZnImCP has a very limited surface area of about 1.1m^2^/g. This should be due to the highly packed structure of the tetrahedral coordination. Hence, ZnImCP is non-porous and in turn, should lessen the photocatalytic degradation process. However, at the same time, any decrease of dyes’ concentrations after water-remediation by ZnImCP under light should be attributed solely to photocatalytic degradation rather than permanent adsorption. Indeed, several works also demonstrated the use of non-porous CP similar materials for photocatalytic degradation [21].

#### TGA analyses of ZnImCP and S-ZnImCPs

**Fig. 6:**
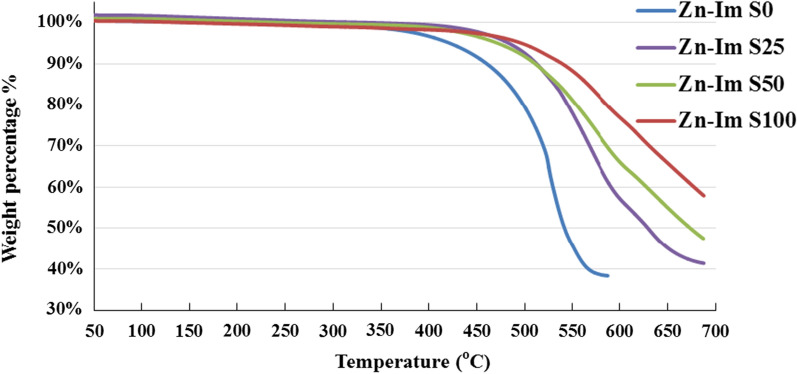
TGA curves for ZnImCP and S-Zn-ImCPs

TGA analyses were performed for ZnImCP and S-ZnImCPs under an inert N_2_ atmosphere with a heating rate of 20 °C min^−1^ in the temperature range from room temperature to 700 °C, and the results are shown in Fig. 6. ZnImCP shows no significant mass loss up to 350 °C. It seems that the sample has no water content. Gradual and smooth decomposition starts at 350 °C and ceased at about 575 °C. The residue mass is about 40% which is in agreement, to some extent, with the percentage of element Zn in ZnImCP (observed 38%, calculated 32%). Similarly, S-ZnImCPs show no significant mass loss, however, up to 450 °C. This indicates that S-treatment supports S-ZnImCPs matrix structures and covalent bonding is suggested. In addition, as sulfur content increases, decomposition becomes less steep and shifts to a higher temperature, i.e. structure becomes more rigid.

For all CPs, the presence of a single decomposition step indicates the intact of one phase structures of imidazole-saturated Zn nodes which matches PXRD analysis results. Also, for all CPs, the mass loss should be related to the decomposition of the organic ligand, imidazole.

#### UV–Vis DRS measurements and band-gap energies

**Fig. 7:**
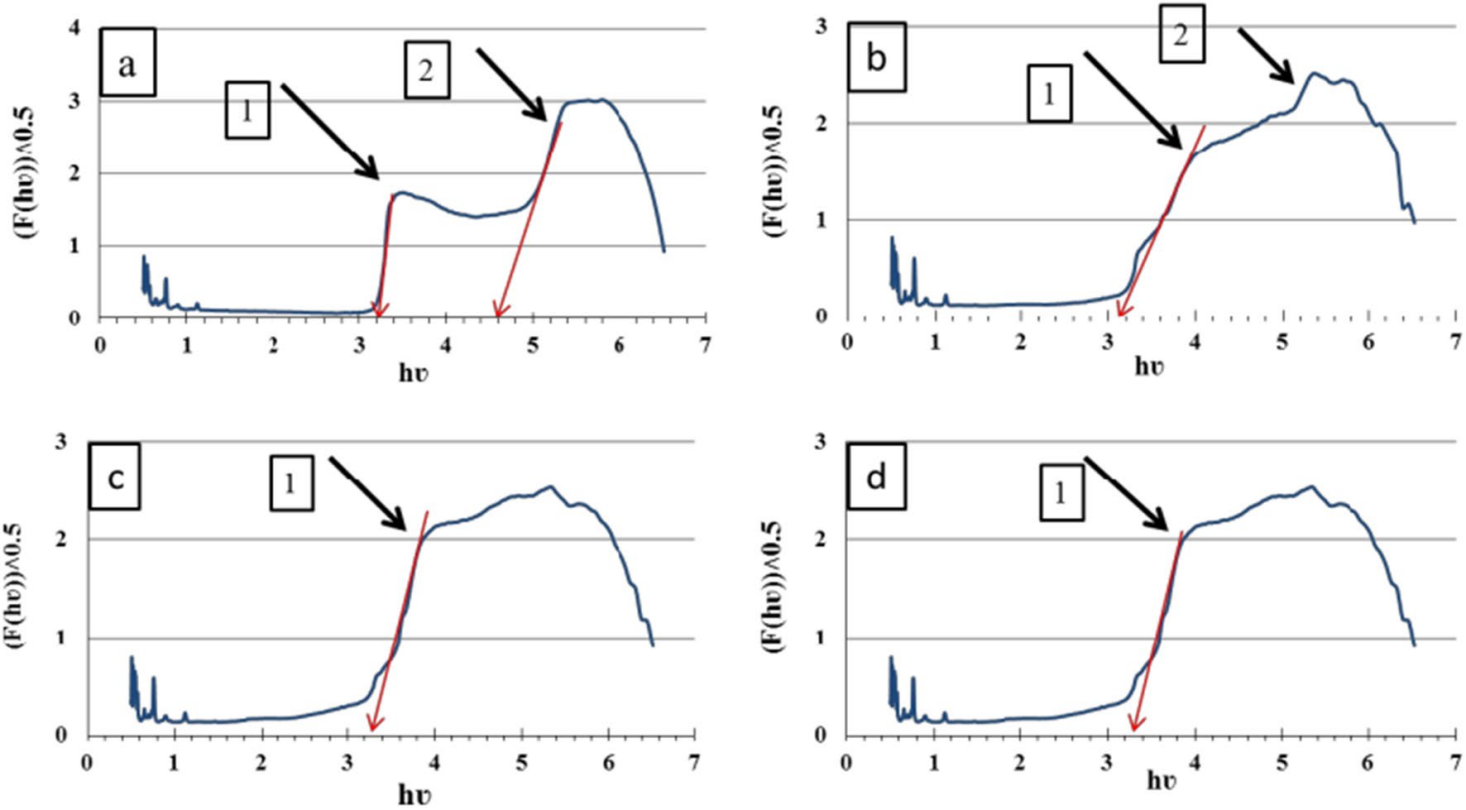
Diffuse reflectance of **a** ZnImCP,**b** 25S-ZnImCP, **c** 50S-ZnIm CP, and **d** 100S-ZnImCP

UV–Vis DRS spectra of ZnImCP and S-ZnImCPs are shown in Fig. 7. First, before discussing the band-gap, it is important to comment on the observation of gradual change of spectrum from the pristine CP towards the 100 ppm CP. ZnImCP has two peaks as shown in Fig. 7 (arrows 1 and 2), and for 25S-ZnImCP the two peaks still present to a certain extent (as shoulders), however for 50S-ZnImCP and 100S-ZnImCP this shoulder flattened. This could be a noteworthy sign of successful chemical incorporation of sulfur into ZnImCP.

Generally, two main mechanisms are known responsible for degradation: (i) the direct mechanism (D-M) or sensitization where radiation excites the dye molecule which upon adsorption by catalyst transfers e^–^ to catalyst’s conduction band, and (ii) the indirect mechanism (InD-M) where radiation excites the catalyst itself elevating e^–^ into the conduction band. *E*_g_ values are assigned in Fig. 7 and Table 4 summarizes these values. ZnImCP has two *E*_g_ values, 4.6 and 3.1 eV. These band-gaps appoint all CPs, pristine and modified, to be InD-M active under UV-light yet inactive under Vis-light. D-M is persistently active as it does not depend on a catalyst *E*_g_.

**Table 4:** *E*_g_ values of ZnImCP and S-ZnImCPs

Sample	*E*_g1_(eV)	*E*_g2_(eV)
ZnImCP	3.10	4.6
25S-ZnImCP	3.10	–
50S-ZnImCP	3.15	–
100S-ZnImCP	3.15	–

It was interesting to find that ZnImCP has two band-gaps a character that is not common, at least for the classical pure photocatalysts. However, the appearance of several band gaps, for some CPs has been mentioned in some recent works. As an example, Dinh Du et al.prepared MIL-101and recorded 1.75, 2.27, and 3.74 eV as three-band gaps, where the absorption band at the UV-region should be contributed by the electron transfer n → π* in terephthalic acid, and the absorption bands in the visible region should be related to 3*d* electron transfer [55]. This implies that multi-band-gaps could originate from inorganic/organic hybrid compounds. Herein, ZnImCP encompasses two inherent *E*_g_ values, 4.6 and 3.1 eV as mentioned. The former value reflects the original imidazole *π* → π* transition as pure imidazole has λ_max_ ≈ 290 nm [56]. The latter could be metal to ligand charge transfer (MLCT) originating upon Zn/imidazolate formation as a ligand to metal charge transfer (LMCT) should not be expected due to metal-*d*^10^ configuration [5, 57].

For the three S-ZnImCPs, each CP has only one *E*_g_ value. The values are 3.1, 3.15 and 3.15 eV for 25S-ZnImCP, 50S-ZnImCP and 100S-ZnImCP,respectively. These values are very close or equal to the lower one of the pristine ZnImCP (3.1 eV) and also reflect the original MLCT transition. Since the higher one (4.6 eV of ZnImCP) has vanished in the three S-ZnImCPs, it should be attributed to the hindrance of imidazolate *π* → π* transition, because of the effective modification by sulfide anion with Zn(II). Three vanishing schemes could be given based on proposed S^2–^ coordination with in-matrix Zn(II): (i) being highly electronegative, the incorporated sulfur atom strongly pulls electron density away from the imidazole-part π system, and consequently fading the π → π* excitation [58], (ii) doping sulfur atom onto ZnImCP creates an intermediate energy level via its *p* orbital and hence, the original π → π* (4.6 eV) weakens or a *p*-level of sulfur atom appears well below the Fermi level [17, 59], and (iii) another possibility is the effect of Zn–S polarity, similar to solvent polarity effect on complexes, where sulfur attracts electrons being more negative and forming positivity on Zn, this stabilizes π* which is often more polar than π, i.e. red shift occurs and hence it can be concluded and anticipated hypsochromic shift upon doping CPs with nonmetal that bond to metal node.

Whatever the vanishing proposal, the vanishing of the 4.6 eV band-gap gives allowance to prop up the excitations via 3.1–3.15 eV band-gaps. It could be highlighted that sites of band-gaps 3.1–3.15 eV could be fortified at the expense of vanished sites of band-gap 4.6 eV. Overall, when considering the InD-M mechanism, all S-ZnImCPs should follow the same behaviour as pristine ZnImCP and should respond solely to UV light via this mechanism.

#### Stability of ZnImCP in Water

**Fig. 8:**
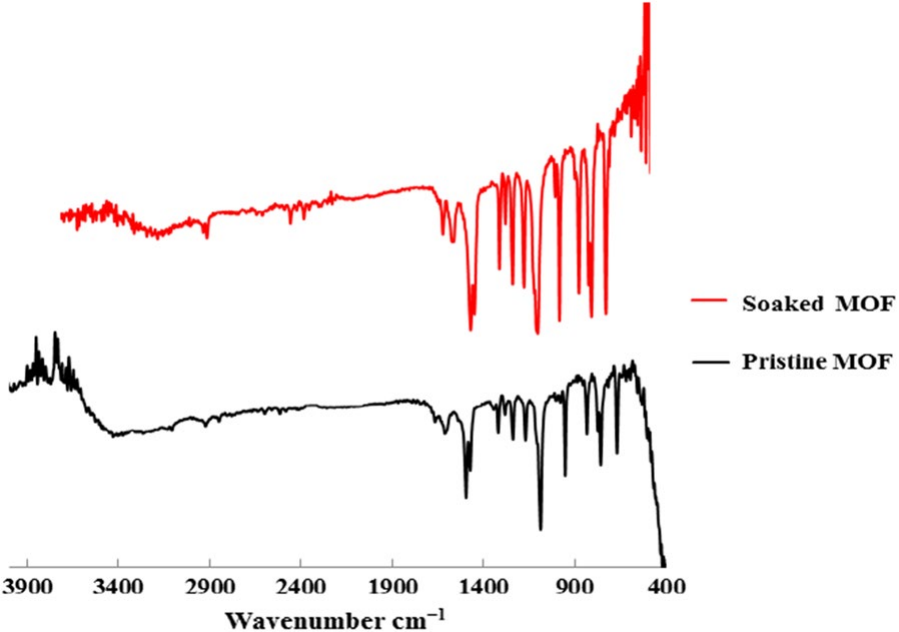
FTIR spectra of the pristine and soaked ZnImCP

A suitable hydro-stability of ZnImCP can be suggested according to the mass loss test. After the first 24 h of water immersion, the mass loss was about 10% and after the next 24 h under the same conditions mass loss was 15%, and then no further mass loss was recorded as the third 24 h showed also 15% mass loss. Moreover, hydro-stability test of 50S-ZnIMCP was tested. The mass loss was about 8% after 24 h immersion. The lower mass loss suggests more hydro-stability upon inclusion of Sulphur. In addition, IR spectra of ZnImCP before and after soaking are almost similar as shown in Fig. 8. This elucidates the chemical stability of ZnImCP against water. The mass loss might be due to: (i) solubility of some non-reacted imidazole or zinc acetate and/or (ii) some partial hydrolysis of non-well coordinated branches.

#### pH_pzc_ of ZnImCP and its buffering behaviour

The recognition of pH value**(s)** at which photocatalysis and photolysis occur is imperative to understanding the degradation mechanisms. Considering photocatalysis, the pH of the solution should have some influence on a catalyst-surface charge, the solvent/medium (H_2_O) and dye structure. Hence, assigning pH_pzc_ helps specify the charge of the material surface, positive or negative, depending on the applied pH. Determination of pH_pzc_ is always applied for classical semiconductors, e.g. TiO_2_ and ZnO [60]. The pH_pzc_ has been determined, as well, for several CPs that have been used in photocatalytic degradation of dyes [61, 62]. The determination of pH_pzc_ is important as it helps in proposing the mechanism of degradation based on the adsorption of ionic dyes on the catalyst surface, i.e. the electrostatic attraction/repulsion between charged dye molecules in solution, H^+^, OH^‒^ ions and the charge of the CP surface [63, 64].

In general the surface of metal oxides, as examples of classical semiconductors, is affected by the pH of the applied solution due to their structural nature where oxygen is responsible for their essential negatively charged surface. And hence, identification of pH_pzc_ should be very fruitful for deducing the degradation mechanism which depends initially on the electrostatic attraction between charged dye molecules and the surface of metal oxides semiconductors. On the other hand, CPs are materials whose structures are different. They include organic linkers comprising, generally, groups such as carboxylates, nitro, hydroxyl and amines. These groups are responsible for controlling the pH of not only the CPs surfaces but also the solution too through interaction with H^+^ and OH^−^. These functional groups could be able to establish the buffering effect that causes the response to pH change [65, 66].

**Fig. 9:**
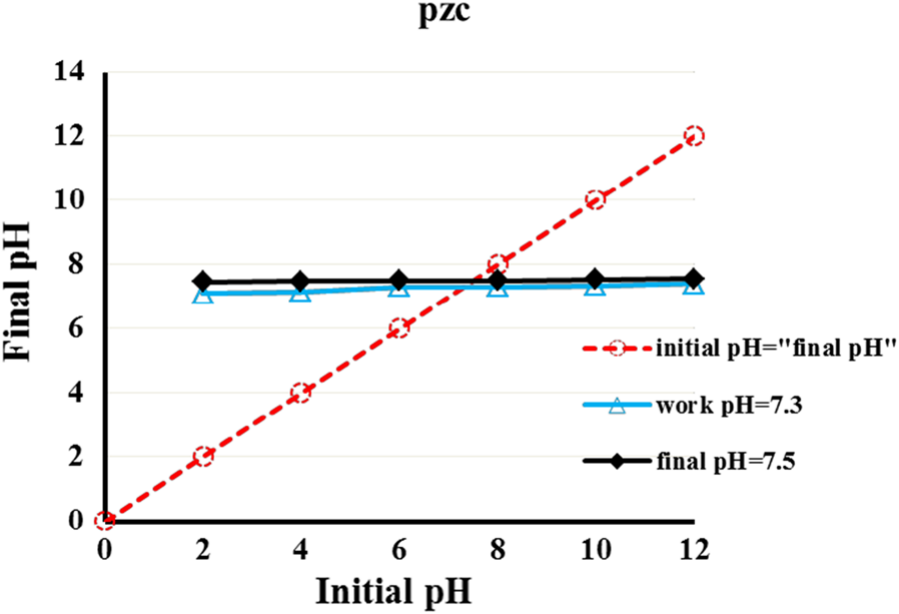
Determination of (pH_pzc_) of ZnImCP

For ZnImCP, Fig. 9 indicates that the measured final pH values for all applied initial pH values were almost the same, about pH 7.5. This value was recorded at equilibrium, i.e. after 24 h of immersing the CP in different solutions of specified initial pH. Though, the more practical pH value is that one measured during the catalysis process, that is during the first 5 h and it was found to be about pH 7.3. However, during the first 60 min, the operating pH slowly changed towards 7.3 for all applied initial pH. In contrast, for blank solution (i.e. for photolysis where CP is not present) the initial pH values did not significantly change from the beginning till the end of the experiments. Hence, the change of the initial pH values upon immersion of the CP to a certain constant one indicates that the response to the initial pH values is essentially related to the effect of ZnImCP with no significant contribution from the dyes.

The value pH 7.3 can be considered as pH_pzc_ for ZnImCP and also reflects the buffering behaviour of the ZnImCP [12, 67]. This property is completely different from that of traditional semiconductors which retain acidity and shows a positively charged surface when treated with an acidic solution retains basicity and show a negatively charged surface when treated with a basic solution.

The buffering behaviour of ZnImCP should originate from its chemical structure. In ZnImCP, Zn(II) is tetrahedrally coordinated with imidazole where both the N-pyridine and N-pyrrole of each imidazole are engaged in coordination. As such, hence, both N-pyridine nor N-pyrrole cannot respond to the applied initial pH. Therefore, it can be suggested that the ZnImCP structure suffers from some defects where some imidazole molecules are coordinated through N-pyridine leaving N-pyrrole coordination-free.

**Fig. 10:**
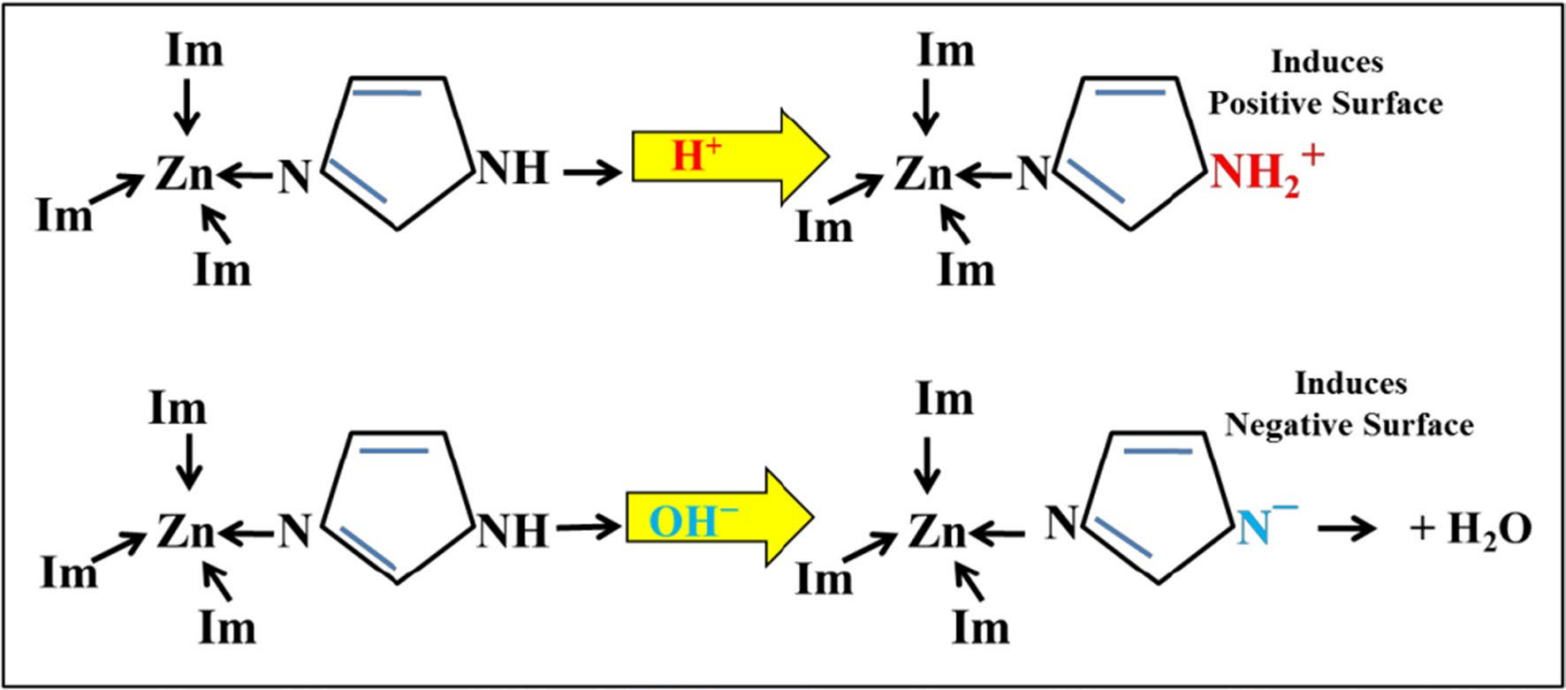
Buffering behaviour of ZnImCP

Figure 10 schematically illustrates the coordination environment of Zn(II) in defected sites of ZnImCP and how it act in response to acidic and basic aqueous media. It is suggested that The − NH moiety should be responsible for the buffering behaviour. In acidic and to some extent in neutral media (pH < 7.3), − NH group responds to acidity, i.e. H^+^, by forming − NH_2_^+^ (− NH/ − NH_2_^+^ pair) with liberating OH^−^ that causes the increase in the solution pH to 7.3. On the other hand, in a basic medium (pH > 7.3), − NH group responds to basicity, i.e. OH^–^, forming − N^–^ (− NH/ − N^–^ pair) and producing H_2_O. These responses should be considered as a buffering act. Thic action have been elucidated in several works, e.g. He et al. designed and synthesized an organic ligand that behaves as buffer guards to enhance the aqueous stability of the synthesized CP (JUC-1000) by preserveing its structural integrity at low and high pH values. They declared that the applied ligand can alter its total charge at high or low pH values due to the presence of the following pairs: − O^−^/ − OH, − OH/ − OH_2_^+^, − NH − / − NH_2_^+^ − and − NH/ − N^−^ [66]. Additionally, some reports showed the acidic and basic behaviour of − CH and –NH moieties as demonstrated by their role in acidic and basic catalysis of some reactions [68–73].

Accordingly, for degradation experiments, it should be kept in mind that the operating pH is about 7.3 whatever the applied initial pH is. In addition, it should be clear that buffering modifies the surface charge of CPs being positive for initially applied acidity whereas being negative for initially applied basicity. In general and as a gain from the previous elucidation, it is recommended that the determination of pH_pzc_ for several CPs/MOFs with similar understanding as for classical semiconductors should be careful as it could be misleading in some cases.

#### Effect of buffering behaviour of ZnImCP on photocatalytic degradation of dyes

Principally, immersing a buffering solid in water solution regulates solution pH in a manner that causes extraordinarily positivity of surface for acidic initial pH and extraordinarily negativity of surface for basic initial pH and both leaving behind almost neutral medium and a highly populated stern layer with counter ions Cl^–^ and Na^+^ (from HCl and NaOH as pH adjusting agent),respectively. Consequently, the effect of buffering behaviour on an aqueous medium should be considered for mechanistic interpretation and should be responsible for some degradation similarities in the present study.

The buffering behaviour of ZnImCP causes changes on its surface when it is immersed in solutions which affect, consequentially, the photocatalytic degradation process. In addition and at the same time, the solutions of different initial pH values would provide the same pH value which also affects the photocatalytic degradation process. In the majority of publications, contaminant-adsorption is considered an essential pre-step for photocatalytic degradation which permits an effective sensitization mechanism to proceed. This in turn depends on the charged surface and also depends on the solution pH [74–77]. For ZnImCP, adsorption experiments in the dark were performed to determine the removal percentages of MB^+^ and MO^−^ dyes at different initial pH values and the results are shown in Table 5. From the table, adsorption is so limited for almost all initial pH values and even null for some. However, remarkable photocatalytic degradations were recorded for all applied initial pH values as given in the table. Hence, the solitary effect of electrostatic attraction/repulsion rule between catalyst surface and ionized dye for effective photocatalysis is therefore under question considering InD-M.

**Table 5:** Adsorption in the dark and photocatalytic degradation of MB^+^ and MO^−^ at different initial pH values

Dye	MB			MO		
Initial pH	4	7	10	4	7	10
Ads.% in dark	null	null	10%	24%	5%	null
*D*% under vis	36%	80%	84%*	10%	10%	2%
Degradation amount (mg)	0.046	0.100	0.105	0.001	0.001	0.003
*D*% under UV	38%^a^ 0.048	52% 0.065	76% 0.095	34% 0.043	35% 0.044	50%^a^ 0.063

^a^Photolysis solely is in action

Accordingly, in the present study, the dye-adsorption mechanism could not be exclusively effective [3, 21]. This is supported by several works. For example, Cui et al. declared that there was no obvious decrease in the absorbance value of MB aqueous solution when admixed with their synthesized CP1 and CP2 in dark environment for 120 min, and they concluded the non-possibility of adsorbing MB molecule into CP1 and CP2 frameworks. Nonetheless, photocatalytic degradations up to 91.4% for CP1 and 89.1% for CP2 were recorded after 120 min of UV-irradiation [3]. In another example, Partha et al.explicated the negligible adsorption of the dyes orange G, RHB, Remazol Brilliant Blue R, and MB, by their applied CPs, yet photocatalytic degradations of these dyes were considerable [78]. These two reports support that adsorption is not an essential necessary pre-step in photocatalytic degradation. Otherwise, the intimate contact (maybe of limited time-duration relative to adsorption) between the ionic dye and charged surface of the catalyst due to weak coulomb forces interactions (some sort of, may be London force) can be considered [79].

#### Photocatalytic activity of ZnImCP and S-ZnImCPs

The photocatalytic activities of ZnImCP and S-ZnImCPs were evaluated by applying degradation of two dyes of a different type as test pollutants, MB (cationic dye, MB^+^) and MO (anionic dye, MO^–^), under Vis-light (35 W, 400–800 nm) and UV-light (24 W, 254 nm), applying different initial pH values (4, 7, and 10) at room temperature.

#### Photocatalytic degradation of MB

##### Under Vis-light

**Fig. 11:**
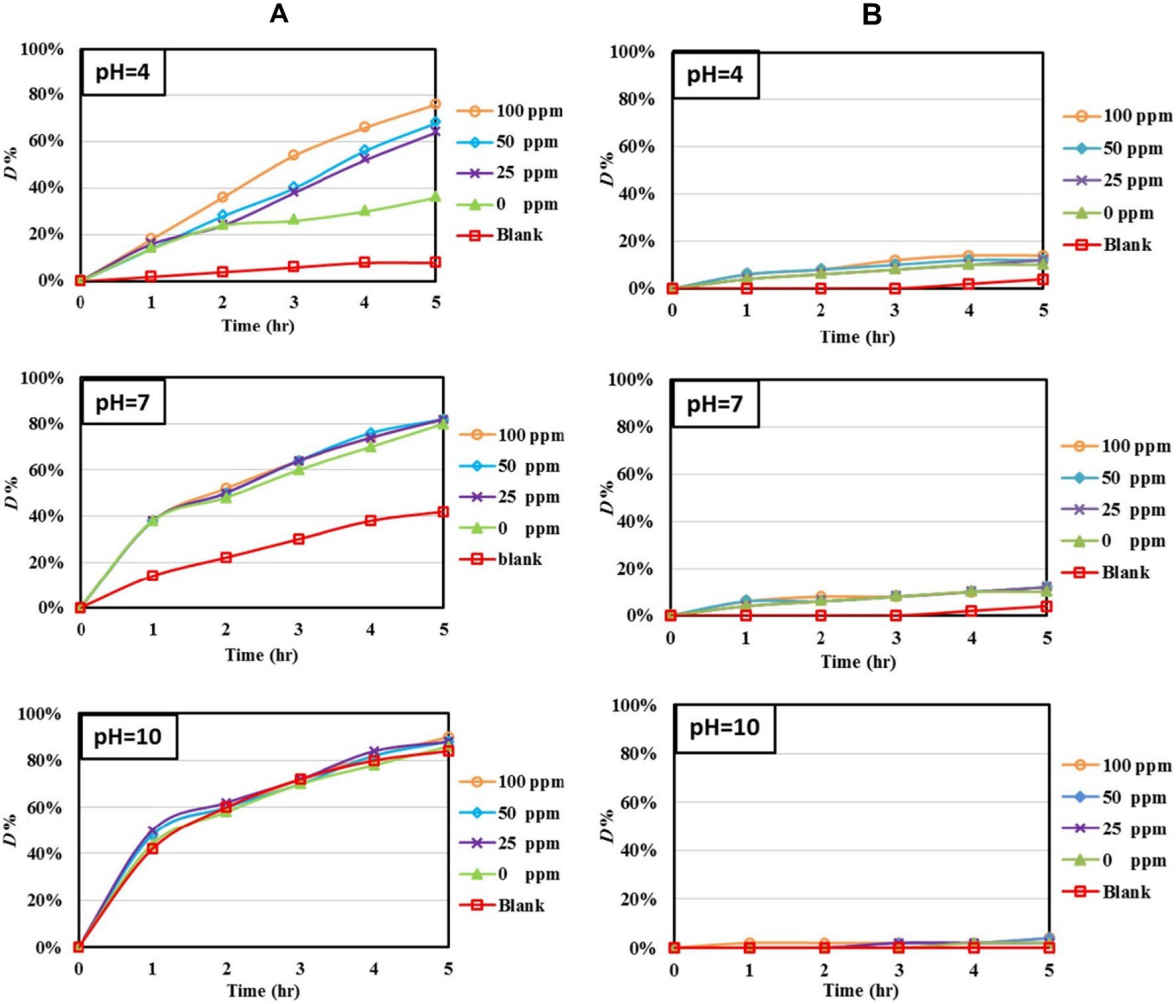
Photocatalytic and photolytic degradations of **A** MB and **B** MO at different pH under Vis-light

Regarding photocatalysis under Vis-light, InD-M is not operating due to the improper band-gap with this applied light and hence only D-M is in operation. Plots of photolytic and photocatalytic degradations with a time of MB by ZnImCP and S-ZnImCPs under Vis-light are presented in Fig. 11(A). Appraising MB-photolysis solely, from the figure, it increases with initial pH. Generally, an increase in pH accelerates photolysis due to further hydroxyl radicals generation [80]. For photocatalysis, it should be mentioned that the observed degradation is the resultant of both photocatalysis and photolysis. The latter should proceed even though the catalyst is present [81]. To itemize the effective photocatalysis, it was calculated by subtracting the photolysis result from the apparent photocatalysis result after 5 h, Table 6.

**Table 6:** Effective *D*% of photocatalyses and the corresponding degraded amounts

Dye	Initial pH	ZnImCP	25S-ZnImCP	50S-ZnImCP	100S- ZnImCP
		*D*%, (amount, mg)			
MB	4	27 (0.034)	59 (0.074)	63 (0.079)	69 (0.086)
	7	37(0.046)	40 (0.050)	40 (0.050)	40 (0.050)
	10	2 (0.003)	3 (0.004)	3 (0.004)	5 (0.006)
MO	4	8 (0.010)	9 (0.011)	10 (0.013)	10 (0.013)
	7	6 (0.008)	7 (0.009)	7 (0.009)	8 (0.010)
	10	2 (0.003)	4 (0.005)	4 (0.005)	4 (0.005)

Hence, from the figure, photocatalysis by ZnImCP increases with initial pH, however, this increase is apparent due to the engagement of photolysis. Hence, effective photocatalytic degradation has been calculated and is given in Table 6. Effective photocatalytic degradation has the increasing order pH 10 (2%) < pH 4 (27%) < pH 7 (37%). This indicates that D-M is nearly prohibited for initial pH 10 even though the surface is strongly negative and should be readily MB^+^-attracting. Herein, buffering functions. Parallel to inducing negative surface (–N^–^ due to buffering behaviour of ZnImCP), an intensively populate Na^+^-stern layer is formed which should effectively screen the MB^+^ adsorption/immediacy with catalyst surface [12]. For initial pH 7, the surface is almost neutral and the stern layer is charge-faded and hence giving the chance of impermanent-adsorption/immediacy of MB with CP surface and hence D-M can play its role. For initial pH 4, photocatalysis is considerable even though the surface is positive due to buffering (being highly populated by –NH_2_^+^), and should repel MB^+^ causing less chance for D-M. However, a highly populated Cl^–^-stern layer gives a chance for MB^+^ to permeate towards CP surface (under shaking conditions), where the probability of certain immediacy to surface increases [12].

Considering S-ZnImCPs, photocatalysis for initial pH 10 is also prohibited similar to that of pristine ZnImCP for the same cause. From Table 6, effective degradation efficiency with initial pH has the increasing order of 10 < 7 < 4 for all S-ZnImCPs. For initial pH 7, photocatalysis is all similar (slightly higher) to that of pristine ZnImCP as the surface is neutral. It seems that doping does not efficiently affect degradation and may be due to the evident strong photocatalysis by ZnImCP. For initial pH 4, sulfur doping is useful where degradation is better than that of ZnImCP and regularly increases with sulfur content. The presence of Zn–S sites represents attractive negative centers (sulfur atoms would favour the attraction of the cationic molecules) and give more chance for MB-adsorption/immediacy and consequently, D-M is possible and that’s why degradation enhances with the increase of sulfur content.

Overall, photocatalytic degradation of MB by of ZnImCP and S-ZnImCPs, under Vis-light, is recommended for neutral ad acidic media.

Photolytic and photocatalytic degradations of MO by ZnImCP and S-ZnImCPs under Vis-light are presented in Fig. 11(B). Considering photolysis, from the figure, degradation is negligible for all initial pH. From Table 6, photocatalytic degradations by ZnImCP and S-ZnImCPs are very partial, yet are about fivefold those of photolysis for initial pH 4 and 7. Photocatalysis for these pH values should follow the sensitization mechanism only. MO does not show similar degradation as MB for all pH values probably because of its narrower absorption band under visible light compared to MB, hence a non-effective self-sensitization mechanism.

##### Under UV-light

**Fig. 12:**
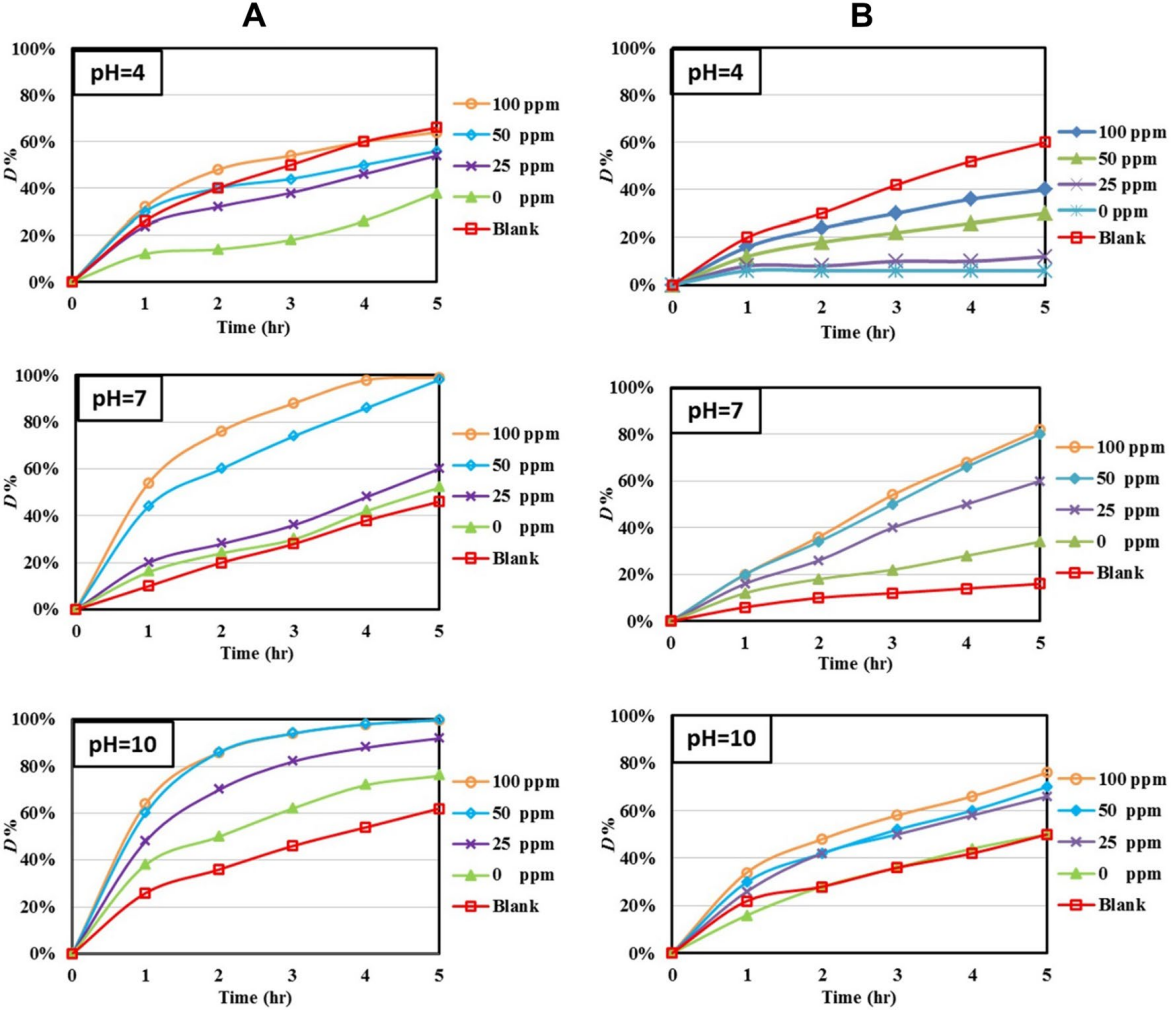
Photocatalytic and photolytic degradations of **A** MB and **B** MO at different pH under UV light

The photolytic and photocatalytic degradations efficiency of MB by ZnImCP and S-ZnImCPs under UV light are presented in Fig. 12(A). Appraising photolysis solely from the figure; shows a considerable effect for all initial pH values. For photocatalysis by ZnImCP and S-ZnImCPs, both the D-M and InD-M should be in action under UV light.

For the initial pH 10, photocatalysis is more effective than photolysis and effectiveness increases as sulfur content increases. Compared with under Vis-light case for the same initial pH, D-M should similarly be prohibited (Na^+^ population); hence only InD-M is in action and is responsible for degradation. Zn–S sites are effective e^–^ collecting centers producing ˙O_2_^–^ which strongly being repelled towards solution bulk, and cause degradation enhancement. For initial pH 7, the order of degradations is almost the same as that for initial pH 10 and both mechanisms are in action where D-M contributes due to the neutral surface. Initial pH 10 shows better degradation than initial pH 7, hence In-D mechanism is the main player where repelled ˙O_2_^–^ migration is an important step.

For the initial pH 4, photocatalysis shows lower values than that of photolysis which means that the presence of CPs depressed the photolysis mechanism. This behaviour is, of course, against process aim willing. However, this is an interesting phenomenon that should be dealt with starting from the assumption that it should be correlated to both, the UV-light and the buffering behaviour.

For diluted aqueous solutions, solutes do not adequately respond to radiation (weakly excited or ionized), and on the other hand, water gives the following final assortment of radiolytic products [82]:

$${\text{H}}_{{2}} {\text{O}} \to {\text{e}}_{{{\text{aq}}}}^{-} ,{\mathbf{HO}}^{ \cdot } ,{\text{H}}^{ \cdot } ,{\text{ HO}}_{{2}}^{ \cdot } ({\text{H}}^{ + } + {}^{ \cdot }{\mathbf{O}}_{{\mathbf{2}}}^{-} ),{\text{ H}}^{ + } \left( {{\text{H}}_{{3}} {\text{O}}^{ + } } \right),{\text{ OH}}^{-} ,\,\,{\mathbf{H}}_{{\mathbf{2}}} {\mathbf{O}}_{{\mathbf{2}}} ,{\text{ H}}_{{2}}$$

The most important degrading radical is the superoxide anion, ˙O_2_^–^. Species like ˙OH and H_2_O_2_ are also oxidizing and should also be considered. However, regarding ˙O_2_^–^, one proposal of photocatalysis depression is based on catalysts buffering that creates an exceedingly positive surface that sturdily attracts˙O_2_^–^ and confines it within the stern layer which hardly diffuses outwards to attack MB in bulk solution. Besides upon excitation, ˙O_2_^–^ should also be produced on the catalyst surface as follows:

$${\text{O}}_{{2\,{\text{dissolved}}}} + {\text{e}}_{{{\text{CB}}}}^{ - } \to^{ \cdot } {\text{O}}_{{\,2\,{\text{ads}}}}^{ - }$$

Under these conditions ˙O_2_^–^ should play its role in degradation, however, the following equation shows the quenching of ˙O_2_^–^ and also conversion of ˙OH and H_2_O_2_ into H_2_O [83]:

$${}^{ \cdot }{\text{O}}_{{2}}^{-} \mathop{\longrightarrow}\limits^{{{\text{e}}_{{{\text{aq}}}}^{-} ,{ 2}{\mathbf{H}}^{ + } }}{\text{H}}_{{2}} {\text{O}}_{{2}} \left( {{\text{stern}}} \right)\mathop{\longrightarrow}\limits^{{{\text{e}}_{{{\text{CB}}}}^{-} }}{}^{ \cdot }{\text{OH }} + {\text{ OH}}^{-} \mathop{\longrightarrow}\limits^{{{\text{e}}_{{{\text{aq}}}}^{-} ,{ 2}{\mathbf{H}}^{ + } }}{\text{2 H}}_{{2}} {\text{O}}$$

As the equation shows, quenching requires an abundance of H^+^ which is achieved by the direct radiolysis of water and for the applied initial pH 4. In addition, the following catalyst-dependent reaction is another continuous source of H^+^ [84]:

$${\text{H}}_{{2}} {\text{O}}_{{{\text{ads}}}} + {\text{ h}}_{{{\text{VB}}}}^{ + } \to {}^{ \cdot }{\text{OH }} + {\text{ H}}^{ + }$$

Attracting attention, as sulfur content increases depression weakens, i.e. photocatalysis recurs. The rate of radicals quenching to H_2_O starts to restrain by extra production of ˙O_2_^–^ on S-ZnImCPs as sulfur becomes a center of e_CB_^–^ congregation.

The photolytic and photocatalytic degradations of MO by of ZnImCP and S-ZnImCPs under UV light are presented in Fig. 12(B). From the figure, photolysis is the least for all pH conditions except in one case, the initial pH 4. This is the same as for MB. Hence, dye charge does not play a role. As mentioned above it should be a matter of ˙O_2_^–^ quenching. Generally for pH 7 and 10, photocatalytic degradation increases and pH 4 depression decreases with sulfur content. Obviously, for all applied initial pH 4, 7 and 10, degradation under UV light is far much more effective than degradation under Vis-light. InD-M is suggested to be dominant here, especially for initial pH 7 and 10. The formed **˙**O_2_^–^ can escape the catalyst surface (being neutral for initial pH 7 and negative for pH 10) towards solution-bulk where degradation redox reaction readily takes place. As a summary, Table 7 shows the effective photocatalyses, *D*%.

**Table 7:** Effective *D*% of photocatalyses and the corresponding degraded amounts

Dye	Initial pH	ZnImCP	25S-ZnImCP	50S-ZnImCP	100S- ZnImCP
		*D*%, (amount, mg)			
MB	4^a^	− 27	− 13	− 10	− 3
	7	3 (0.004)	13 (0.016)	53 (0.066)	53 (0.066)
	10	16 (0.020)	30 (0.038)	38 (0.048)	38 (0.048)
MO	4^a^	− 56	− 48	− 30	− 20
	7	15 (0.019)	42 (0.053)	61 (0.076)	64 (0.084)
	10	0 (0.000)	20 (0.025)	23 (0.029)	26 (0.033)

^a^ photolysis depression by CPs

##### The re-use of ZnImCP

**Fig. 13:**
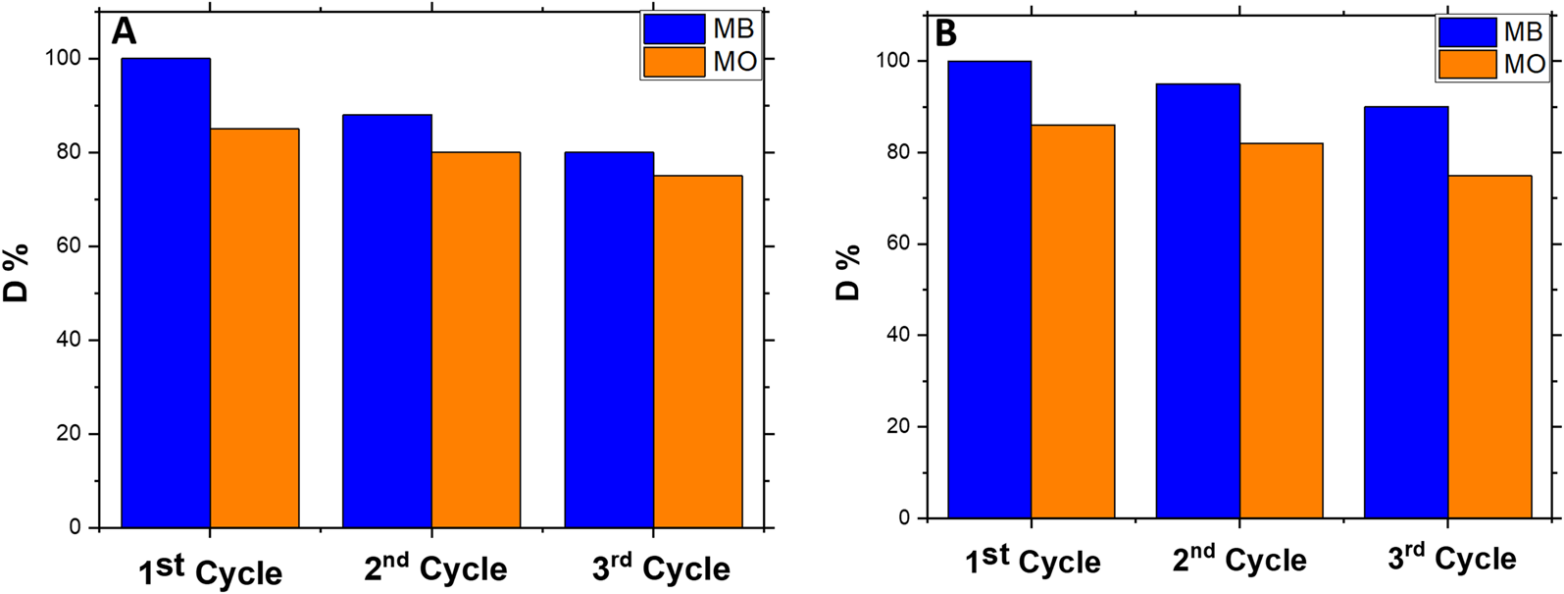
Photocatalytic degradation of MB and MO using pristine ZnImCP **A**, and 50S-ZnImCP **B** at different cycling runs under UV-light at pH = 7

The hydro-stability of the ZnImCP provides the possibility of successive uses. Consequently, the reusability of the ZnImCP was evaluated by applying the same degradation experiment consecutive 3 times as a preliminary assessment. A sample of ZnImCP was used for MB degradation under UV light at pH 7 three consecutive times, Fig. 13a. shows the result. The third experiment shows degradation less than the first one by about 10%. Table 8 shows the re-use comparison between ZnImCP and some other CP catalysts for MB only. The same process was performed yet with MO and Fig. 13a shows the result. The third experiment shows degradation less than the first one by about 12%. 50S-ZnImCP also show good reusability after three consecutive. The third cycle shows degradation less than the first one by about 5% as shown in Fig. 13b.

**Table 8:** Comparison between re-use of ZnImCP and other CPs with MB at pH = 7, under UV light

CP/ no. of cycles (MB)	*D*% (normalized)		
	Cycle 1	Cycle 2	Cycle 3
ZnImCP [this work]	100%	88%	80%
Fe-doped copper MOF [85]	100%	99%	98.5%
Co CP [3]	100%	94%	91%

## Conclusions

ZnImCP has been facilely synthesized in an aqueous solution at low temperature (70 °C) and simply doped by sulfur forming S-ZnImCPs. These CPs showed a typical photo-responsive property where the pristine CP has two band-gaps while modified CPs are of a single band-gap. These CPs are also solid buffering materials which can amend the pH of the contaminant aqueous solutions near to its pH_pzc_ whatever the applied initial pH. ZnImCP and S-ZnImCP are crystalline supporting their photocatalysis behaviour. The doped sulfur is suggested to bond with Zn(II) nodes which enhances thermal stability and modifies the band-gap. Under Vis-light and specifically for initial pH 4, sulfur doping is effective for higher degradation of MB and regularly increases with sulfur content where Zn–S sites represent attractive negative centers giving more chance for MB-adsorption/immediacy and D-M operation. Under UV-light and specifically for initial pH 7 and 10, both MB and MO degradations are enhanced as sulfur content increases and the In-D mechanism is suggested to be in action. The degradation-depression effect of ZnImCP on photolysis is observed for the initial pH 4 under UV light for both MB and MO and this depression weakens as sulfur content increases.

## Data Availability

The datasets used or analysed during the current study are available from the corresponding author on reasonable request.
